# Antiretroviral therapy and HIV‐associated cardiovascular disease: a prospective cardiac biomarker and CMR tissue characterization study

**DOI:** 10.1002/ehf2.14603

**Published:** 2023-12-15

**Authors:** Pieter‐Paul S. Robbertse, Anton F. Doubell, Tonya M. Esterhuizen, Philip G. Herbst

**Affiliations:** ^1^ Division of Cardiology, Department of Medicine, Faculty of Medicine and Health Sciences Stellenbosch University and Tygerberg Hospital Cape Town South Africa; ^2^ University of Pittsburgh HIV‐Comorbidities Research Training Programme in South Africa Cape Town South Africa; ^3^ Division of Epidemiology and Biostatistics, Department of Global Health Stellenbosch University Stellenbosch South Africa

**Keywords:** Cardiac biomarkers, Soluble ST2, Galectin‐3, HIV‐associated cardiovascular disease, Cardiovascular magnetic resonance imaging, Antiretroviral therapy

## Abstract

**Aims:**

Biochemical markers are fundamental in cardiac evaluation, and various novel assays have recently been discovered. We prospectively evaluated the hearts of newly diagnosed people living with human immunodeficiency virus (PLWH) using cardiac biomarkers, compared them with human immunodeficiency virus (HIV)‐uninfected controls, and correlated our prospective findings with cardiovascular magnetic resonance imaging (CMR).

**Methods and results:**

Newly diagnosed, antiretroviral therapy (ART)‐naïve PLWH were recruited along with HIV‐uninfected, age‐matched, and sex‐matched controls. All participants underwent measurement of high‐sensitivity cardiac troponin T (hs‐cTnT), N‐terminal pro‐B‐type natriuretic peptide (NT‐proBNP), soluble ST2 (sST2), and galectin‐3, as well as a CMR study with multiparametric mapping. The HIV group started ART and was re‐evaluated 9 months later. The cardiac biomarkers and their correlation with CMR parameters were evaluated in and between groups. Compared with controls (*n* = 22), hs‐cTnT (4.0 vs. 5.1 ng/L; *P* = 0.004), NT‐proBNP (23.2 vs. 40.8 ng/L; *P* = 0.02), and galectin‐3 (6.8 vs. 9.0 ng/mL; *P* = 0.002) were all significantly higher in the ART‐naïve group (*n* = 73). After 9 months of ART, hs‐cTnT (5.1 vs. 4.3 ng/L; *P* = 0.02) and NT‐proBNP (40.8 vs. 28.5 ng/L; *P* = 0.03) both decreased significantly and a trend of decrease was seen in sST2 (16.5 vs. 14.8 ng/L; *P* = 0.08). Galectin‐3 did not demonstrate decrease over time (9.0 vs. 8.8 ng/mL; *P* = 0.6). The cardiac biomarkers that showed the best correlation with CMR measurements native T1, T2, and extracellular volume were NT‐proBNP (*r*_*s*_ ≥ 0.4, *P* < 0.001) and galectin‐3 (*r*_*s*_ ≥ 0.3, *P* < 0.01).

**Conclusions:**

Our cardiac biomarker data support the presence of subclinical myocardial injury, remodelling, and fibrosis at HIV diagnosis, and ART had a positive influence on these blood markers. It remains unclear if the underlying pathological processes were fully addressed by ART. The ability of cardiac biomarkers to detect and track tissue abnormalities diagnosed with CMR showed promise. With additional research, this could lead to improvements in screening and monitoring myocardial abnormalities, even in CMR‐limited settings.

## Introduction

The association between human immunodeficiency virus (HIV) infection and cardiovascular dysfunction and mortality is well known.^1^ Despite advances and the increased availability of antiretroviral therapy (ART), people living with HIV (PLWH) remain at higher risk for cardiovascular disease (CVD).^2^ The profile of CVD differs between persons on ART and those not on ART.^3^, ^4^, ^5^, ^6^ In high‐income countries, coronary artery disease, myocardial infarction, and heart failure are frequently reported complications of HIV infection.^3^, ^7^ Research from low‐ and middle‐income countries suggests that HIV‐associated cardiomyopathy (HIVAC) remains a significant contributor to myocardial disease in sub‐Saharan Africa,^3^, ^8^, ^9^ the region that has the highest burden of HIV infection.^10^

Mounting evidence from cardiovascular magnetic resonance imaging (CMR) studies suggests that the hearts of HIV‐positive individuals may already be functionally and structurally abnormal at the time of HIV diagnosis.^11^, ^12^, ^13^, ^14^ CMR has been the tool of choice to study the various pathological processes considered to play a part in the clinical syndrome of HIVAC.^4^ However, CMR research from the African continent is under‐represented^4^, ^15^ and not widely available in resource‐limited settings due to high cost and limited expertise. Studies utilizing alternative modalities alongside CMR are required to identify surrogates of imaging biomarkers.

Cardiac biomarkers have proven invaluable in the evaluation of the cardiovascular system in research and clinical practice. Numerous cardiac biomarkers have been described and are already utilized in routine clinical practice. These may be broadly categorized as markers of myocardial injury/stress, neurohormonal activation, and cardiac remodelling.^16^

High‐sensitivity cardiac troponin T (hs‐cTnT), a marker of myocardial injury, is frequently detectable in chronic heart failure, and even low detectable values are closely linked to adverse clinical outcomes.^17^ Left ventricular (LV) wall stretch from increased pressure or volume is a potent stimulus for the secretion of B‐type natriuretic peptide (BNP). BNP is evidenced by the presence of the prohormone, N‐terminal pro‐BNP (NT‐proBNP), a benchmark biomarker to which other biomarkers are compared,^16^ and routinely employed in diagnosing acute and chronic heart failure. Soluble ST2 (sST2) and galectin‐3 are novel biomarkers of cardiac remodelling.^16^ sST2 is involved with LV hypertrophy, fibrosis, and remodelling,^18^ and galectin‐3 is thought to be directly involved with ventricular remodelling via tissue repair and collagen deposition.^16^ sST2 has prognostic value but, unlike BNP, is not affected by age, renal function, and body mass index (BMI)^16^ and has been shown to predict future heart failure, even in healthy persons.^19^

We set out to test two hypotheses in this study. Firstly, we hypothesize that the cardiac biomarkers of HIV‐infected persons are raised compared with HIV‐uninfected persons, reflecting subclinical CVD. Secondly, we hypothesize that CMR myocardial imaging biomarkers correlate with biochemical cardiac biomarkers hs‐cTnT, NT‐proBNP, sST2, and galectin‐3 in a newly diagnosed group of PLWH.

## Methods

### Study design and participants

Detailed methodology regarding the recruitment process has been published elsewhere.^13^ The original cohort was expanded upon^14^ and analysed in this study. Briefly, newly diagnosed, HIV‐infected individuals (referred to as the naïve group) were recruited into a prospective cohort study in the Western Cape, South Africa, alongside a healthy, HIV‐uninfected control group that was frequency matched for age and sex. The study complies with the Declaration of Helsinki and was approved by the Stellenbosch Human Research Ethics Committee (Ref. S19/07/137), and all participants provided written informed consent. The HIV status of all persons was confirmed with a serum HIV enzyme‐linked immunosorbent assay, and participants were enrolled before the initiation on first‐line ART. All patients aged 18–55 years, without known current or prior cardiac disease and without ART use prior to enrolment, and who were not pregnant or acutely unwell (including current coronavirus disease 2019) were deemed eligible for inclusion. Potential participants with a contraindication to CMR or gadolinium contrast use were excluded. Participants diagnosed with tuberculosis (TB) coinfection were not excluded. HIV/TB‐coinfected participants were enrolled at least 2 weeks after initiation on TB treatment while still ART naïve. All treatment and workup provided to HIV‐infected participants were in line with the South African treatment guidelines.^20^

### Clinical data collection and follow‐up

**Figure 1:**
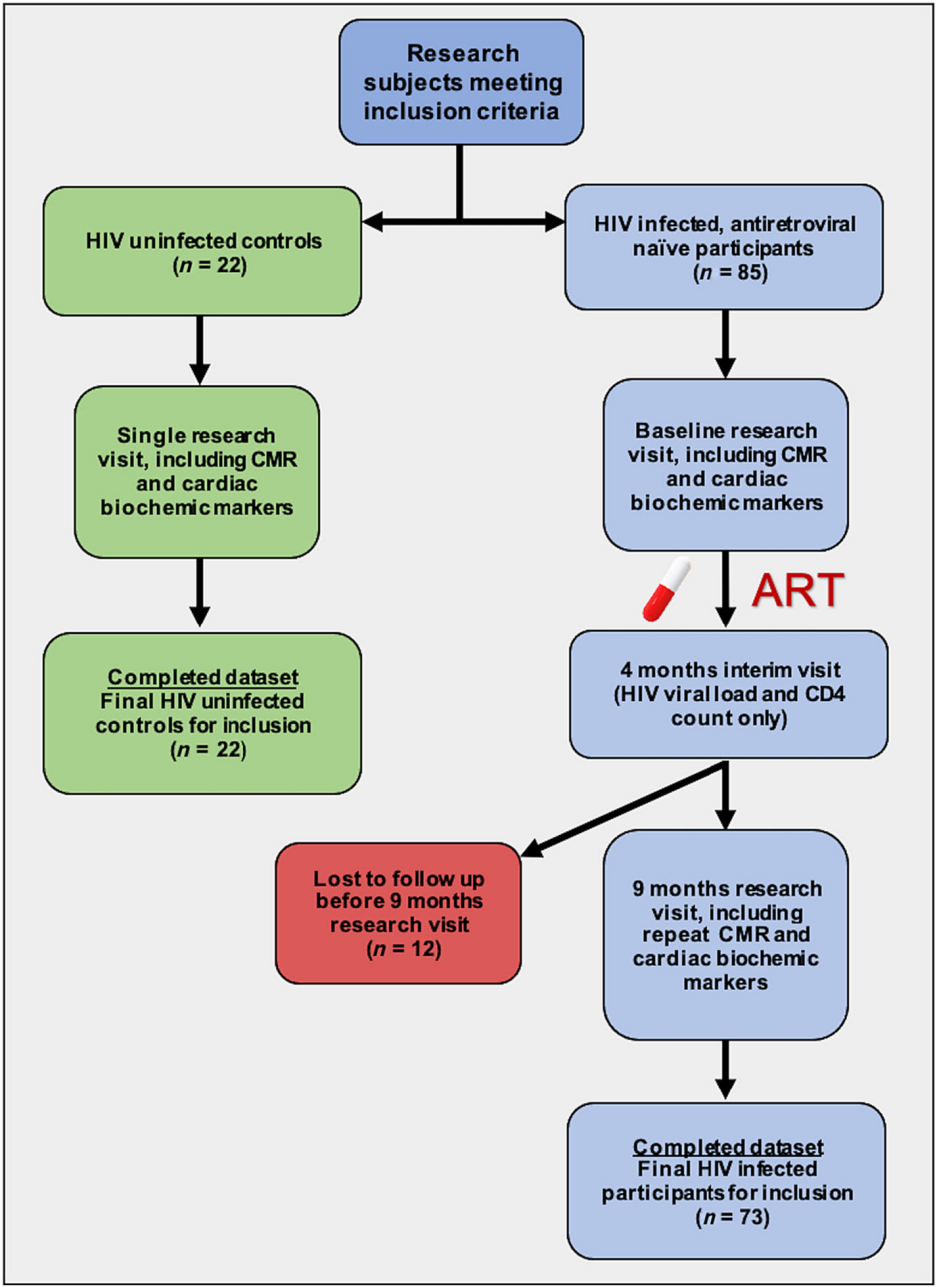
Study flow chart. ART, antiretroviral therapy; CMR, cardiovascular magnetic resonance imaging; HIV, human immunodeficiency virus.

The naïve group was seen at a baseline visit prior to ART and a final follow‐up after at least 9 months on ART (referred to as the ART group), while the controls had a once‐off research visit (see *Figure* *1*). All research participants were seen in the morning following a fast of at least 10 h. A full clinical evaluation including anthropometric, biochemical, immunological, virological, and CMR investigations was performed at both visits. The World Health Organization (WHO) HIV clinical stage was documented.^21^ Height and weight were measured, and BMI was calculated. Fasting blood samples were analysed by the on‐site National Health Laboratory Service (ISO 15189‐accredited laboratory)^22^ for measurement of urea and electrolytes, glucose, blood lipids, full blood count, differential cell count, HIV enzyme‐linked immunosorbent assay, high‐sensitivity C‐reactive protein (hs‐CRP), hs‐cTnT (Elecsys systems, Roche Diagnostics, Mannheim, Germany), NT‐proBNP (Elecsys systems, Roche Diagnostics, Mannheim, Germany), flow cytometry‐based CD4‐ and CD8 count, and HIV‐1 viral load. To further evaluate hs‐cTnT and NT‐proBNP, the upper limit of normal was defined as 14 and 125 ng/L, respectively, in accordance with biochemical studies using these specific assays.^23^ The linear range for the measurement of HIV viral load was 20–10 000 000 copies/mL or 1.3–7 log (Abbott Alinity m HIV‐1 assay, Abbott, Illinois, USA). Gender‐specific estimated glomerular filtration rate (eGFR) was calculated using the Cockcroft–Gault formula.^20^

### Novel cardiac biomarkers soluble ST2 and galectin‐3

Blood samples collected at baseline and final follow‐up were centrifuged and stored at −80°C for batch analysis of novel biomarkers sST2 and galectin‐3. All analyses were performed by the Stellenbosch University Immunology Research Group (ISO 15189‐accredited laboratory).^22^ Serum concentrations were determined using multiplexed immunometric assays (Human Magnetic Luminex Screening Assay, R&D Systems, Minneapolis, USA).

### Cardiovascular magnetic resonance

#### Image acquisition and analysis

As previously described,^13^, ^14^ CMR studies were performed on a single 1.5 T magnetic resonance scanning system (Magnetom Avanto, Siemens Healthcare, Germany) with commercially available cardiac sequences. Studies were performed using standard methods and imaging planes.^24^ Breath‐held, electrocardiogram (ECG)‐gated, balanced steady‐state free precession (bSSFP) cine images were obtained for the assessment of cardiac mass, volumes, function, and morphological evaluation. To evaluate the myocardial tissue characteristics, native T1 and post‐contrast T1 mapping, T2 mapping, and late gadolinium enhancement (LGE) images were acquired. P.‐P.S.R. performed unblinded post‐processing and analysis on all the CMR data using cvi42 (Version 5.11.2, Circle Cardiovascular Imaging, Calgary, Alberta, Canada). Semi‐automated, artificial intelligence‐assisted endocardial and epicardial LV and right ventricular (RV) borders were traced at end‐systole and end‐diastole, excluding the papillary muscles, to determine ventricular volumes and LV mass. For quantitative multiparametric mapping measurements, the LV endocardial and epicardial borders were traced in basal, mid, and apical short axis to acquire mean values on native T1, T2, and extracellular volume (ECV) maps. Short‐axis ECV maps were created with post‐processing software using the following formula:

$$\mathrm{ECV}=\left(1-\text{haematocrit}\right)\frac{\frac{1}{\text{post}-\text{contrast}\;T1\;\text{myocardium}}}{\frac{1}{\text{post}-\text{contrast}\;T1\;\text{blood pool}}}-\frac{\frac{1}{\text{native}\;T1\;\text{myocardium}}}{\frac{1}{\text{native}\;T1\;\text{blood pool}}}$$

LGE was qualitatively evaluated.

### Statistical analysis

This study was nested within a larger project that evaluated the function, morphology, and tissue characteristics as primary outcomes in HIV‐infected persons using CMR.^13^, ^14^ The biomarker analysis formed part of the original study design and was deemed explorative. The sample size calculation was performed for the CMR‐measured LV volumes, LV mass, and LV ejection fraction (LVEF) using paired‐samples *t*‐test for the prospective evaluation of the HIV‐infected group. Statistical analysis was performed using SPSS Version 27 (IBM Corporation, New York). Continuous data are presented as mean ± standard deviation. For non‐normally distributed data, the median and inter‐quartile range are reported. Due to the highly skewed nature of the cardiac biomarker data, the geometric means ± geometric standard deviations are reported. Categorical data are presented as frequencies with percentages. The normality of data was visually assessed using histograms and normal quantile plots and formally tested using the Shapiro–Wilk test. For cross‐sectional analyses between the HIV‐infected and control groups, the *χ* ^2^ test, independent‐samples *t*‐test, and Mann–Whitney *U* test were used as appropriate. For the prospective analysis of the HIV‐infected group, the paired‐samples *t*‐test, Wilcoxon signed‐rank test, and McNemar test were employed as appropriate. Where statistical significance was borderline or additional information was sought, independent‐samples *t*‐tests and paired‐samples *t*‐tests were performed on non‐normally distributed parameters following log transformation. Bivariate correlations were calculated using Spearman's rho. The changes in the biochemical parameters over time (HIV‐infected group) were correlated with the changes in the tissue characterization parameters native T1, T2, and ECV to evaluate if the direction of the change was similar. Additional explorative bivariate correlations of biochemical markers and cardiac parameters were performed as well. Statistically significant correlations with *r*_*s*_ ≥ 0.2 are reported as these analyses are explorative and hypothesis generating in nature. Statistical significance was two‐tailed and defined as a *P* value ≤0.05.

## Results

### Study group characteristics

The study groups' characteristics and clinical data are shown in *Table* *1*. The naïve group (*n* = 73) and controls (*n* = 22) were well matched in terms of age and sex. Both groups were relatively young with a mean age of 33 and 32 years, respectively. The income of the naïve group was considerably lower compared with controls and reflects the lower socio‐economic status of this population. Although the naïve group had more smokers (49% compared with 27% in the control group), this did not reach statistical significance. No participant had a history of injection drug use. Chronic disease and medication use were rare and comparable between the controls and the naïve group. Two participants from the naïve group had a known diagnosis of hypertension. During follow‐up, one HIV‐infected participant developed hypertension and received treatment. Metabolic syndrome was rare with only one control and two HIV‐infected participants fulfilling the National Cholesterol Education Program Adult Treatment Panel III^25^ criteria at enrolment.

**Table 1:** Study population

Parameter	Control group (*n* = 22) (controls)	HIV‐infected ART‐naïve (*n* = 73) (naïve)	*P* value^a^	HIV‐infected 9 months on ART (*n* = 73) (ART)	*P* value^b^
Age (years)	33 ± 7	32 ± 7	0.6	—	—
Female sex	11 (50%)	33 (45%)	0.6	—	—
Monthly household income (South African rand per month)					
<5000	4 (18%)	53 (73%)	**0.02**	—	—
5000–20 000	14 (77%)	19 (26%)	—	—	—
>20 000	4 (18%)	1 (1%)	—	—	—
Smoking history	6 (27%)	36 (49%)	0.07	37 (51%)	—
Ethanol (units per week)	0.5 (0–7)	4 (0–12)	0.2	0 (0–11)	**0.01**
Waist circumference (cm)	95 ± 18	80 ± 9	**<0.001**	82 ± 10	0.06
Body mass index (kg/m^2^)	30 ± 8	23 ± 4	**<0.001**	24 ± 4	**<0.001**
World Health Organization HIV clinical stage					
I	—	28 (38%)	—	28 (38%)	—
II	—	19 (26%)	—	19 (26%)	—
III	—	25 (34%)	—	24 (34%)	—
IV	—	1 (1%)	—	2 (1%)	—
On treatment for TB	—	10 (14%)	—	2^c^ (3%)	—
Pulmonary	—	8	—	0	—
Extrapulmonary	—	2	—	2	—
Medications					
Salbutamol metred‐dose inhaler	1 (5%)	1 (1%)	—	1 (1%)	—
Rifampicin/isoniazid/pyrazinamide/ethambutol	—	10 (14%)	—	2 (3%)	—
Trimethoprim/sulfamethoxazole	—	21 (29%)	—	14 (19%)	—
Isoniazid prophylaxis	—	1 (1%)	—	43 (59%)	—
Pyridoxine	—	10 (14%)	—	43 (59%)	—
Tenofovir/lamivudine/dolutegravir	—	Naïve	—	71 (97%)	—
Losartan	—	—	—	2 (3%)	—
Amlodipine/hydrochlorothiazide	—	1 (2%)	—	3 (4%)	—
Statins	—	—	—	1 (1%)	—
Blood pressure (mmHg)					
Systolic	119 ± 13	112 ± 16	0.07	112 ± 15	0.7
Diastolic	76 ± 9	71 ± 10	**0.04**	68 ± 10	**0.02**
Mean arterial pressure	90 ± 9	85 ± 11	**0.04**	83 ± 11	0.1

ART, antiretroviral therapy; HIV, human immunodeficiency virus; TB, tuberculosis.

Continuous variables are mean ± standard deviation or median (inter‐quartile range) unless otherwise specified. *P* values in bold indicate statistical significance.

^a^ Controls vs. naïve.

^b^ Naïve vs. ART.

^c^ One additional patient developed tuberculosis after recruitment.

### The general health of the human immunodeficiency virus‐infected group

Sixty‐six per cent of the cohort had early (WHO Stage I or II) disease. A third of the naïve group visited their health centre due to significant, unexplained weight loss and were otherwise well. The remainder of the naïve group presented due to contact tracing or with HIV‐related complications. These included dermatological manifestations of HIV, lymphadenopathy, and respiratory, gastrointestinal, and miscellaneous complaints. HIV/TB coinfection was found to be prevalent among newly diagnosed PLWH. Ten participants from the naïve group (14% of the group) had TB disease diagnosed around the time of enrolment. All but one of the TB cases was mild and managed on an outpatient basis. Extrapulmonary TB was diagnosed incidentally in two cases at enrolment (abdominal and pleural TB, respectively). No participant reported any significant loss of functional capacity or dyspnoea.

### Novel cardiac biomarkers and biochemical and immunological data

The novel cardiac biomarker data are shown in *Table* *2*. The geometric mean hs‐cTnT of the naïve group at baseline was elevated compared with controls (*P* = 0.004). Eight ART‐naïve participants (11% of the group) had raised hs‐cTnT levels. None of the controls had elevated hs‐cTnT. The naïve group had a median NT‐proBNP of more than double that of the control group (*P* = 0.02). Nine ART‐naïve participants (13% of the group) had raised NT‐proBNP compared with one case in the control group. Compared with the control group, novel cardiac marker galectin‐3 was higher in the naïve group (*P* = 0.002). Relatively increased levels of sST2 were demonstrable in the naïve group as well, although statistical significance was not reached (*P* = 0.1).

**Table 2:** Cardiac biomarkers

Parameter	Control group (*n* = 22) (controls)	HIV‐infected ART‐naïve (*n* = 73) (naïve)	*P* value^a^	HIV‐infected 9 months on ART (*n* = 73) (ART)	*P* value^b^	Change (Δ) in HIV group over time (*n* = 73)
hs‐cTnT (ng/L)	4.0 ± 1.3	5.1 ± 2.1	**0.004**	4.3 ± 1.5	**0.02**	0 (−1.0 to 0.8)
NT‐proBNP (ng/L)	23.2 ± 2.3	40.8 ± 2.9	**0.02**	28.5 ± 2.5	**0.03**	−10.0 (−36.5 to 22.3)
sST2 (ng/mL)	13.4 ± 1.5	16.5 ± 1.7	**0.11**	14.8 ± 1.7	0.08	−2.2 (−6.5 to 1.4)
Galectin‐3 (ng/mL)	6.8 ± 1.2	9.0 ± 1.5	**0.002**	8.8 ± 1.3	0.6	0.1 (−1.4 to 1.6)

ART, antiretroviral therapy; HIV, human immunodeficiency virus; hs‐cTnT, high‐sensitivity cardiac troponin T; NT‐proBNP, N‐terminal pro‐B‐type natriuretic peptide; sST2, soluble ST2.

Continuous data are presented as geometric mean ± standard deviation. Change in parameters (ART value minus naïve value) is presented as median (first quartile to third quartile). *P* values in bold indicate statistical significance.

^a^ Controls vs. ART‐naïve group compared with log_10_‐transformed variables.

^b^ ART‐naïve group vs. ART group compared with log_10_‐transformed variables.

The geometric mean hs‐cTnT of the ART group was lower at the final follow‐up compared with the naïve group (*P* = 0.02). Furthermore, all cases with raised hs‐cTnT at enrolment normalized after 9 months on ART (*P* = 0.006). The geometric mean NT‐proBNP decreased by 30% at the final follow‐up (*P* = 0.03). In addition, when evaluating cases with raised NT‐proBNP, a significant decrease was seen after the initiation on ART (nine cases at baseline vs. three cases at final follow‐up; *P* < 0.001). Galectin‐3 did not demonstrate change when evaluating the ART group. sST2 showed a trend to decrease at the final visit, although statistical significance was not reached (*P* = 0.08).

Biochemical and immunological data are displayed in *Table* *3*. A marked decrease in the median hs‐CRP was observed when comparing the naïve group and the ART group (*P* = 0.01). At 9 months, most of the ART groups showed viral suppression with a median viral load of 20 copies/mL.

**Table 3:** Biochemical and immunological evaluation

Parameter	Control group (*n* = 22) (controls)	HIV‐infected ART‐naïve (*n* = 73) (naïve)	*P* value^a^	HIV‐infected 9 months on ART (*n* = 73) (ART)	*P* value^b^	Change (Δ) in HIV group over time (*n* = 73)
Serum creatinine (μmol/L)	72 ± 14	70 ± 14	0.6	82 ± 16	**<0.001**	13 ± 12
eGFR (mL/min/1.73 m^2^)	111 ± 38	92 ± 22	**0.03**	83 ± 19	**<0.001**	−9 ± 21
Blood lipids (mmol/L)						
Total cholesterol	4.3 (3.8–5.0)	3.4 (2.9–4.0)	**<0.001**	3.5 (3.0–4.1)	0.1	1 (−0.3 to 0.5)
LDL	2.7 (1.9–3.2)	1.8 (1.5–2.4)	**0.004**	1.9 (1.6–2.4)	0.9	−0.03 (−0.3 to 0.4)
HDL	1.4 (1.2–1.5)	1.0 (0.8–1.3)	**0.001**	1.2 (1.0–1.4)	**<0.001**	0.2 (−0.1 to 0.4)
TG	0.9 (0.6–1.9)	0.8 (0.7–1.2)	0.8	0.7 (0.6–1.0)	**0.03**	−0.1 (−0.4 to 0.1)
hs‐CRP (mg/L)	2.4 (1.1–9.2)	3.4 (0.8–13.7)	0.5	1.9 (0.5–7.5)	**0.01**	−0.5 (−7.8 to −0.5)
CD4 count (cells/μL)	—	289 (170–410)	—	378 (271–593)	**<0.001**	100 (26–195)
CD8 count (cells/μL)	—	817 (584–1147)	—	624 (446–858)	**<0.001**	−122 (−393 to 54)
CD4:CD8 ratio	—	0.35 (0.19–0.45)	—	0.60 (0.39–0.94)	**<0.001**	−0.1 (−0.6 to 0.6)
HIV viral load (log copies/mL)	—	4.9 (4.0–5.5)	—	1.3^c^ (1.3–1.6)	**<0.001**	−3.4 (−4.1 to −2.4)

ART, antiretroviral therapy; eGFR, estimated glomerular filtration rate; HDL, high‐density lipoprotein; HIV, human immunodeficiency virus; hs‐CRP, high‐sensitivity C‐reactive protein; LDL, low‐density lipoprotein; TG, triglycerides.

Continuous data are presented as mean ± standard deviation, geometric mean ± standard deviation (where indicated), or median (first quartile to third quartile). *P* values in bold indicate statistical significance.

^a^ Controls vs. ART‐naïve group.

^b^ ART‐naïve group vs. ART group.

^c^ Twenty copies/mL (log = 1.3) is the lower limit of measurement for our HIV‐1 assay.

In the naïve group, no difference in persons with and without current TB coinfection was evident for cardiac biomarkers hs‐cTnT, sST2, and galectin‐3 (*P* > 0.2). In persons with TB disease, the median NT‐proBNP was seen to be lower compared with persons that did not have TB coinfection [15.5 ng/L (inter‐quartile range: 10.0–41.0) vs. 44.5 ng/L (inter‐quartile range: 19.8–85.0)] (*P* = 0.03).

### Cardiac morphology, function, and tissue characterization

The CMR parameters evaluating cardiac morphology, function, and tissue characterization are shown in *Table* *4*. Both the global native T1 and T2 mapping values were higher in the naïve group compared with controls (*P* > 0.02). The mean global ECV of the control group measured 2% lower compared with the naïve group.

**Table 4:** CMR cardiac morphology, function, and tissue characterization

Parameter	Control group (*n* = 22) (controls)	HIV‐infected ART‐naïve (*n* = 73) (ART‐naïve)	*P* value^a^	HIV‐infected 9 months on ART (*n* = 73) (ART)	*P* value^b^	Change (Δ) in HIV group over time (*n* = 73)
LA area (cm^2^)	20.0 ± 3.1	20.2 ± 3.9	0.8	21.0 ± 3.8	**0.05**	0.7 ± 3.1
RA area (cm^2^)	18.9 ± 3.1	19.6 ± 3.8	0.4	20.4 ± 3.4	**0.01**	0.7 ± 2.5
LV mass indexed to height (g/m)	60 (51–71)	63 (53–73)	**0.05** ^c^	63 (53–74)	0.8	4 (−3 to 11)
LV end‐diastolic volume indexed to height (mL/m)	81 ± 13	86 ± 16	**0.03** ^d^	89 ± 15	**0.008**	4 ± 12
LV sphericity index	0.53 ± 0.04	0.53 ± 0.05	0.6	0.52 ± 0.04	0.4	0 ± 0.03
LV ejection fraction (%)	63 ± 5	60 ± 6	**0.03**	59 ± 5	0.6	0 ± 5
Global native T1 mapping (ms)	1008 ± 31	1032 ± 44	**0.02**	1014 ± 34	**<0.001**	−18 ± 38
Global T2 mapping (ms)	46 ± 1.6	48 ± 2.7	**0.006**	48 ± 2.1	0.5	0 ± 2
Global ECV index (%)	24 ± 2.8^e^	26 ± 4.1	0.1	25 ± 3.1	**0.001**	−1.5 ± 3.7
LGE present	1 (10%)^e^	35 (49%)	**0.02**	40 (55%)	0.2	—
Pattern of LGE						
Subepicardial	—	18 (50%)	—	18 (45%)	0.7	—
Midmyocardial	1 (100%)^e^	12 (33%)	—	17 (43%)	—	—
Mixed	—	6 (17%)	—	5 (12%)	—	—

ART, antiretroviral therapy; CMR, cardiovascular magnetic resonance imaging; ECV, extracellular volume; HIV, human immunodeficiency virus; LA, left atrial; LGE, late gadolinium enhancement; LV, left ventricular; RA, right atrial.

Continuous variables are mean ± standard deviation or median (inter‐quartile range) unless otherwise specified. *P* values in bold indicate statistical significance.

^a^ Controls vs. ART naïve group

^b^ ART‐naïve group vs. ART group.

^c^ Mean difference = 6 g when corrected for age, sex, race, estimated glomerular filtration rate, and current tuberculosis.

^d^ Mean difference = 6 mL when corrected for age, sex, race, ethanol use, and current tuberculosis.

^e^ Fifty per cent of the controls received gadolinium contrast (*n* = 11).

After ART initiation, significant change in the myocardial mapping was observed when evaluating the ART group. The global native T1 and global ECV showed significant change towards normalization and were statistically similar in the ART group when compared with the control group (*P* = 0.5). No significant change was observed in the global T2 after ART initiation. LGE was highly prevalent in the naïve group. Predominantly subepicardial and midmyocardial LGE was seen at the basal segments. The prevalence and pattern of LGE did not differ significantly in the naïve group and ART group (*P* > 0.2).

### Correlation of cardiac biomarkers with study parameters

The correlation coefficients of parameter change [ART group's value minus naïve group's value (delta, Δ)] are shown in Supporting Information, *Appendix* *S1*. ΔNT‐proBNP and Δgalectin‐3 demonstrated a positive correlation with Δhs‐CRP (*r*_*s*_ = 0.2, *P* = 0.04, and *r*_*s*_ = 0.4, *P* = 0.003, respectively). Furthermore, both these biomarkers showed a positive correlation with Δglobal native T1 (*r*_*s*_ = 0.5, *P* = 0.001, respectively) and Δglobal ECV (*r*_*s*_ = 0.5, *P* < 0.001, and *r*_*s*_ = 0.3, *P* = 0.008, respectively). ΔNT‐proBNP correlated with Δgalectin‐3 (*r*_*s*_ = 0.4, *P* = 0.003), and ΔsST2 correlated with Δgalectin‐3 (*r*_*s*_ = 0.5, *P* < 0.001). ΔHIV viral load correlated with Δhs‐cTnT (*r*_*s*_ = 0.3, *P* = 0.04), ΔNT‐proBNP (*r*_*s*_ = 0.3, *P* = 0.003), and Δgalectin‐3 (*r*_*s*_ = 0.3, *P* = 0.006).

When evaluating all participants with HIV infection (the naïve and ART groups), additional explorative correlations were performed. A full list of the bivariate correlations within the HIV‐infected participants is shown in Supporting Information, *Appendix* *S2*. NT‐proBNP and galectin‐3 demonstrated the strongest correlation with tissue mapping and were as follows: NT‐proBNP and global native T1 (*r*_*s*_ = 0.4), global T2 (*r*_*s*_ = 0.5), and global ECV (*r*_*s*_ = 0.4) (*P* < 0.001); galectin‐3 and global native T1 (*r*_*s*_ = 0.3, *P* = 0.003), global T2 (*r*_*s*_ = 0.3, *P* < 0.001), and global ECV (*r*_*s*_ = 0.3, *P* < 0.001). The cardiac biomarkers that showed the strongest correlation with LV mass, LV end‐diastolic volume, and RV end‐diastolic volume were hs‐cTnT (*r*_*s*_ = 0.3, *P* < 0.001, respectively) and sST2 (*r*_*s*_ = 0.3, *P* = 0.002, respectively). HIV viral load (log) demonstrated a positive correlation with NT‐proBNP, sST2, and galectin‐3 (*r*_*s*_ = 0.2, *P* < 0.03).

In the naïve group (*n* = 73), the right atrial (RA) size showed a weak negative correlation with galectin‐3 (*r*_*s*_ = −0.3, *P* = 0.02), and LV mass showed a weak positive correlation with sST2 (*r*_*s*_ = 0.3, *P* = 0.02). Neither hs‐cTnT, NT‐proBNP, sST2, nor galectin‐3 demonstrated a significant correlation with the left atrial (LA) size, LV end‐diastolic volume, RV end‐diastolic volume, LVEF, or RV ejection fraction (RVEF) at baseline.

## Discussion

History has taught us that the tertiary manifestations of disease are often significantly more complex than their primary pathologies. In the last century, we discovered that treatments for various late‐stage infectious diseases such as syphilis and TB did not meaningfully alter disease progression and prognosis.^26^ Early detection and intervention are paramount to better the odds of favourable long‐term outcomes.

Our research is the first to provide prospective data on both cardiac biomarkers and CMR from an ART‐naïve group of PLWH.^13^, ^14^ Our cohort provides data to better understand the biochemical evidence of subclinical CVD in newly diagnosed PLWH and their transition into ART‐experienced persons. Furthermore, our cohort better represents women and people living in sub‐Saharan Africa compared with the current literature.

Our key findings were as follows:

- At the time of HIV diagnosis, hs‐cTnT, NT‐proBNP, and the novel cardiac biomarker galectin‐3 were elevated in HIV‐infected persons compared with healthy age‐ and sex‐matched persons.
- After 9 months of ART, hs‐cTnT and NT‐proBNP were observed to decrease significantly in HIV‐infected persons.
- Galectin‐3 levels remained elevated after 9 months on ART.
- At the time of HIV diagnosis, sST2 showed a trend towards elevation in HIV‐infected persons compared with controls. At 9 months of ART, sST2 showed a trend towards decrease. The sST2 observations mirror the cardiac biomarkers hs‐cTnT and NT‐proBNP but did not reach statistical significance.
- The cardiac biomarker findings mirror the CMR observations in that structural, functional, and tissue characterization abnormalities were already present at the time of HIV diagnosis and were influenced by the initiation of ART.
- The Δ cardiac biomarkers that showed the best correlation with Δ myocardial imaging markers native T1, T2, and ECV were NT‐proBNP and galectin‐3, indicating similar directions of change after 9 months of ART.

### Subclinical myocardial disease

The study's data support our hypothesis that the cardiac biomarkers of HIV‐infected persons at the time of diagnosis differ from their HIV‐uninfected peers. As markers of myocardial injury, stretch, and remodelling were all raised, this likely indicates the presence of subclinical pathology. Our previously described CMR tissue characteristics from this cohort further support this notion and provide robust imaging evidence of subclinical myocardial oedema and replacement fibrosis.^14^ The presence of subclinical myocardial disease at the time of HIV diagnosis is the earliest time point at which cardiac abnormalities may be diagnosed. In addition, our cohort provides evidence that the hearts of HIV‐infected persons can demonstrate abnormality in the absence of ART. The prevailing theory is that cardiac inflammation, set in motion by a complex interplay of patient, environmental, immunological, and viral factors, drives myocardial dysfunction and remodelling.^4^, ^7^, ^27^ At what time point these factors are initiated and have their most deleterious effects on cardiovascular health is not well understood, although mounting evidence suggests this happens shortly after HIV seroconversion and before the initiation of ART.

Cross‐sectional work from South Africa in ambulatory, ART‐experienced persons has demonstrated that almost a third of PLWH have raised NT‐proBNP levels.^28^ In addition, raised BNP was significantly associated with not receiving ART at the time of study. Contemporary, prospective cardiovascular research evaluating ART‐naïve PLWH is limited. Data from persons during acute HIV infection (*n* = 49) show distinct similarities to our work.^23^ In this cohort of almost exclusively male patients from a high‐income country, biomarkers NT‐proBNP and hs‐cTnT were elevated shortly after HIV seroconversion and were seen to decrease after viraemic control was achieved (*P* < 0.001).^23^ Further parallels with our work include the association of NT‐proBNP with high HIV viral load and low CD4 count.

Our research provides evidence that the novel cardiac biomarkers sST2 and galectin‐3 correlate with the HIV viral load. As these two biomarkers have been linked to cardiac remodelling and fibrosis,^16^ this hints to the direct or indirect influence of HIV on causing structural changes to the heart as described in this cohort previously.^13^ Similar to NT‐proBNP, a well‐described and robust cardiac biomarker, changes in galectin‐3 over time were seen to correlate with the changes in the HIV viral load over time. Furthermore, change in galectin‐3 correlated well with change in NT‐proBNP itself, suggesting possible clinical utility.

### Cardiac biomarkers and cardiovascular magnetic resonance imaging tissue characterization

Our study's findings support our second exploratory hypothesis that cardiac biomarkers correlate with imaging biomarkers obtained with CMR. We were able to demonstrate correlation of NT‐proBNP and galectin‐3, both in the HIV group as a whole (the naïve and ART groups) and in the prospective changes of parameters in the naïve group. NT‐proBNP showed the best correlation with global native T1, T2, and ECV. Notably, the hs‐cTnT and NT‐proBNP decreased significantly after 9 months in HIV‐infected patients, while galectin‐3 levels remained high after 9 months of ART. On the other hand, among the cardiac biomarkers, NT‐proBNP and galectin‐3 showed the highest correlation with CMR tissue characterisation markers (native T1, T2, and ECV). This suggests that while biomarkers do reflect imaging markers, a gap exists between cardiac biomarkers and imaging markers in their changes over time and further research is clearly required. However, our work provides novel evidence that biochemical cardiac markers may serve as surrogates or adjuncts for CMR tissue characterization, utilized as tissue imaging biomarkers. Using biochemical cardiac markers as a proxy may prove especially useful in resource‐limited settings where CMR services are limited. However, the applicability may be wider than this. In setting with readily available CMR, longitudinal follow‐up may preferentially be done with biochemical cardiac biomarkers after the initial diagnostic fingerprinting with CMR. Furthermore, as more insight is gained into persons at greatest risk for HIVAC, these markers may serve as future adjuncts to diagnose and monitor CVD.

### Predictors of future cardiovascular disease

In the setting of heart failure in HIV‐uninfected persons, NT‐proBNP and hs‐cTnT are strong predictors of clinical outcomes.^16^ Although less extensively studied, higher levels of the novel biomarkers sST2 and galectin‐3 have been linked to adverse cardiovascular outcomes.^29^, ^30^ NT‐proBNP has been shown to predict the future risk of CVD in the general population,^19^ and an incremental increase in sST2 in persons with normal LVEF increased the 5 year risk for systolic dysfunction.^31^ It would not be unreasonable to extrapolate these findings from the general population to PLWH and would suggest increased cardiovascular risk of the HIV group, already present at the time of HIV diagnosis. Considering the already increased cardiovascular risk of PLWH, NT‐proBNP, or other cardiac biomarkers combined with NT‐proBNP may provide additional information to risk stratify PLWH. Biochemical markers associated with systemic inflammation and enhanced coagulation specifically predict CVD in HIV‐infected persons.^32^ As the primary objective of our study was to demonstrate the presence of an underlying pathological process in otherwise healthy PLWH using biomarkers, we unfortunately do not have clinical outcome data to comment on the mid‐ to long‐term predictive value of the cardiac biomarkers. The true prognostic value of cardiac biomarkers in PLWH with or without subclinical CVD will require further study. Important components of possible future risk prediction are clearly emerging and are likely to rely on multimodal cardiac evaluation that may include imaging biomarkers, cardiac biomarkers, and functional surrogates of cardiovascular risk, such as aortic stiffness.^33^ The best combination to predict cardiovascular risk using these parameters will require long‐term follow‐up studies.

Our biomarker findings, further supported by our CMR data from this cohort,^13^, ^14^ are consistent with myocardial injury and remodelling. Although ART had an overall positive effect, it is not clear if the underlying pathological processes were partially or fully terminated. Our explorative study was not designed to allow for confident commentary on the residual difference of the cardiac biomarkers of the ART group and controls. The differences between the groups are small, and a larger sample size will be required to provide better insight. However, we have made the observation that galectin‐3 showed no interim change after 9 months of ART and remained significantly elevated compared with healthy controls. Furthermore, hs‐cTnT, NT‐proBNP, and sST2 all remained marginally elevated compared with healthy controls. This requires further study.

Additional limitations require discussion. The distribution of the hs‐cTnT data proved challenging. Several participants in the naïve group had considerably higher values than the rest of the group, causing marked positive skewing of the dataset. These data points were largely from the upper 25% of the dataset, and it was these values that were seen to decrease substantially over time. Only evaluating the median and inter‐quartile range may lead to an erroneous conclusion, as no numeric change in the median was seen. However, when reporting the geometric mean and comparing the log‐transformed values, change over time is evident.

There is still some uncertainty regarding the study's sST2 findings. The comparison of sST2 in the naïve group and controls was not able to prove elevated levels with a high degree of certainty. In light of sST2 showing a trend to decrease at 9 months and demonstrating a similar pattern as hs‐cTnT, NT‐proBNP, galectin‐3, and CMR tissue characteristics at baseline, it is likely that the sST2 findings reflect true difference between the naïve group and controls. A larger sample size would have improved our ability to compare the three research groups.

As with all observational research, even strong correlation does not necessarily indicate causation. We set out to prospectively study a group of persons in the early stages of HIV, largely free from the confounding effects of cardiotoxic medications and cardiovascular comorbidities, while striving to recruit an unbiased group of PLWH as they present in a real‐world, outpatient setting. Associations within the dataset are therefore accurate and should inform future research questions to disentangle the complexities of CVD in PLWH. Although limited evidence from this cohort suggests that TB and its treatment did not have clear detrimental structural and functional effects on the heart,^13^ our sample size of HIV/TB coinfection is small. Dedicated work to evaluate TB and TB medications' effect on cardiac biomarkers will be required.

The true duration of HIV infection after HIV seroconversion, despite extensive history taking and clinical evaluation, remains an educated guess. This limitation is not unique to our work and has been discussed extensively elsewhere.^33^ In keeping with our clinical experience and current literature, our cohort is an adequate representation of the average new diagnosis of HIV in South Africa.^34^

## Conclusions

In the absence of known CVD, cardiac biomarkers hs‐cTnT, NT‐proBNP, galectin‐3, and likely sST2 as well were increased at the time of HIV diagnosis. Supported by concomitant CMR findings, this suggests that subclinical myocardial injury, remodelling, and fibrosis are present during early HIV infection. After ART initiation, most of the cardiac biomarkers were seen to decrease in the short term, while galectin‐3 did not. This may indicate ongoing myocardial remodelling and fibrosis in PLWH, despite ART and viral suppression. The ability of cardiac biomarkers to detect and track tissue abnormalities diagnosed with CMR deserves further study. These findings may improve our ability to screen for and monitor myocardial abnormalities, even in settings where CMR availability is limited.

## Conflict of interest

None declared.

## Funding

P.‐P.S.R. was supported in part by a Fogarty International Center HIV Research Training Program grant, National Institutes of Health, to the University of Pittsburgh and Stellenbosch University (D43TW010937), SUNHEART and the Harry Crossley Foundation.

## Supporting information


**Appendix S1.** Bivariate correlations of parameter change over time in the HIV‐infected participants (final follow value up minus baseline value [delta value]).
**Appendix S2.** Bivariate correlations within the HIV‐infected group (baseline and follow up).
