# Supplementary material for: Antiretroviral therapy and HIV‐associated cardiovascular disease: a prospective cardiac biomarker and CMR tissue characterization study
Source: ESC Heart Fail. 2023 Dec 15;11(2):748–58. doi: 10.1002/ehf2.14603 (PMC10966217; doi:10.1002/ehf2.14603)
Supplement: Supplementary file 1 — Appendix S1. Bivariate correlations of parameter change over time in the HIV‐infected participants (final follow value up minus baseline value [delta value]). Appendix S2. Bivariate correlations within the HIV‐infected group (baseline and follow up). [file EHF2-11-748-s001.pdf]

# Supplement A: Bivariate correlations of parameter change over time in the HIV-infected participants (final follow value up minus baseline value [delta value])

**Correlations**

| | | | Δhs-cTnT (ng/l) | ΔNT-proBNP (ng/l) | ΔsST2 (ng/ml) | ΔGalectin-3 (ng/ml) | ΔEthanol (units/week) | ΔWeight (kg) | ΔMean arterial pressure (mmHg) | ΔHeart rate (beats/min) | ΔeGFR (ml/min/ 1.73 $m^2$) |
|---|---|---|---|---|---|---|---|---|---|---|---|
| Spearman's rho | Δhs-cTnT (ng/l) | Correlation Coefficient | 1.000 | .110 | .140 | .007 | .006 | -.113 | -.261* | -.005 | -.108 |
| | | Sig. (2-tailed) | . | .357 | .239 | .951 | .959 | .342 | .026 | .965 | .364 |
| | | N | 73 | 72 | 73 | 73 | 73 | 73 | 73 | 73 | 73 |
| | ΔNT-proBNP (ng/l) | Correlation Coefficient | .110 | 1.000 | .076 | .346** | -.069 | -.359** | -.100 | .049 | -.066 |
| | | Sig. (2-tailed) | .357 | . | .526 | .003 | .563 | .002 | .402 | .682 | .583 |
| | | N | 72 | 72 | 72 | 72 | 72 | 72 | 72 | 72 | 72 |
| | ΔsST2 (ng/ml) | Correlation Coefficient | .140 | .076 | 1.000 | .501** | .143 | -.315** | -.193 | .354** | -.285* |
| | | Sig. (2-tailed) | .239 | .526 | . | .000 | .226 | .007 | .101 | .002 | .015 |
| | | N | 73 | 72 | 73 | 73 | 73 | 73 | 73 | 73 | 73 |
| | ΔGalectin-3 (ng/ml) | Correlation Coefficient | .007 | .346** | .501** | 1.000 | .132 | -.367** | -.085 | .446** | -.135 |
| | | Sig. (2-tailed) | .951 | .003 | .000 | . | .265 | .001 | .474 | .000 | .255 |
| | | N | 73 | 72 | 73 | 73 | 73 | 73 | 73 | 73 | 73 |
| | ΔEthanol (units/week) | Correlation Coefficient | .006 | -.069 | .143 | .132 | 1.000 | -.047 | -.051 | .018 | -.026 |
| | | Sig. (2-tailed) | .959 | .563 | .226 | .265 | . | .690 | .667 | .877 | .828 |
| | | N | 73 | 72 | 73 | 73 | 73 | 73 | 73 | 73 | 73 |
| | ΔWeight (kg) | Correlation Coefficient | -.113 | -.359** | -.315** | -.367** | -.047 | 1.000 | .367** | -.240* | .193 |
| | | Sig. (2-tailed) | .342 | .002 | .007 | .001 | .690 | . | .001 | .041 | .101 |
| | | N | 73 | 72 | 73 | 73 | 73 | 73 | 73 | 73 | 73 |
| | ΔMean arterial pressure (mmHg) | Correlation Coefficient | -.261* | -.100 | -.193 | -.085 | -.051 | .367** | 1.000 | -.291* | .354** |
| | | Sig. (2-tailed) | .026 | .402 | .101 | .474 | .667 | .001 | . | .013 | .002 |
| | | N | 73 | 72 | 73 | 73 | 73 | 73 | 73 | 73 | 73 |
| | ΔHeart rate (beats/min) | Correlation Coefficient | -.005 | .049 | .354** | .446** | .018 | -.240* | -.291* | 1.000 | -.128 |
| | | Sig. (2-tailed) | .965 | .682 | .002 | .000 | .877 | .041 | .013 | . | .280 |
| | | N | 73 | 72 | 73 | 73 | 73 | 73 | 73 | 73 | 73 |
| | ΔeGFR (ml/min/1.73$m^2$) | Correlation Coefficient | -.108 | -.066 | -.285* | -.135 | -.026 | .193 | .354** | -.128 | 1.000 |
| | | Sig. (2-tailed) | .364 | .583 | .015 | .255 | .828 | .101 | .002 | .280 | . |
| | | N | 73 | 72 | 73 | 73 | 73 | 73 | 73 | 73 | 73 |
| | ΔHIV viral load (log copies/ml) | Correlation Coefficient | .246* | .278* | .189 | .321** | .080 | -.180 | -.222 | .248* | -.011 |
| | | Sig. (2-tailed) | .036 | .018 | .109 | .006 | .504 | .129 | .059 | .034 | .924 |
| | | N | 73 | 72 | 73 | 73 | 73 | 73 | 73 | 73 | 73 |
| | ΔCD4 count (cells/μl) | Correlation Coefficient | -.114 | -.130 | -.086 | -.128 | .008 | .197 | .059 | -.378** | .019 |
| | | Sig. (2-tailed) | .339 | .275 | .470 | .280 | .947 | .095 | .620 | .001 | .875 |
| | | N | 73 | 72 | 73 | 73 | 73 | 73 | 73 | 73 | 73 |
| | ΔLV EF (%) | Correlation Coefficient | -.062 | -.129 | -.194 | -.193 | .016 | .063 | .041 | -.130 | .143 |
| | | Sig. (2-tailed) | .604 | .279 | .100 | .101 | .893 | .596 | .728 | .274 | .228 |
| | | N | 73 | 72 | 73 | 73 | 73 | 73 | 73 | 73 | 73 |
| | ΔLV EDV for height (ml/m) | Correlation Coefficient | -.091 | .224 | -.375** | -.322** | .028 | .322** | .370** | -.541** | .261* |
| | | Sig. (2-tailed) | .445 | .058 | .001 | .005 | .813 | .005 | .001 | .000 | .026 |
| | | N | 73 | 72 | 73 | 73 | 73 | 73 | 73 | 73 | 73 |

| | | | | | | | | | | | |
|---|---|---|---|---|---|---|---|---|---|---|---|
| | ΔSTIR SIR | Correlation Coefficient | .076 | -.036 | -.012 | .031 | .140 | .059 | -.055 | -.173 | .029 |
| | | Sig. (2-tailed) | .525 | .765 | .920 | .796 | .241 | .620 | .646 | .146 | .810 |
| | | N | 72 | 71 | 72 | 72 | 72 | 72 | 72 | 72 | 72 |
| | ΔEGE SIR | Correlation Coefficient | -.017 | .097 | -.086 | -.025 | .167 | -.002 | .083 | -.385** | -.011 |
| | | Sig. (2-tailed) | .888 | .423 | .471 | .832 | .161 | .988 | .486 | .001 | .925 |
| | | N | 72 | 71 | 72 | 72 | 72 | 72 | 72 | 72 | 72 |
| | ΔGlobal native T1 (ms) | Correlation Coefficient | .075 | .478** | .152 | .520** | .024 | -.252* | -.206 | .469** | -.060 |
| | | Sig. (2-tailed) | .532 | .000 | .203 | .000 | .840 | .032 | .082 | .000 | .620 |
| | | N | 72 | 71 | 72 | 72 | 72 | 72 | 72 | 72 | 72 |
| | ΔGlobal T2 (ms) | Correlation Coefficient | .179 | .382** | -.145 | .043 | -.232* | -.039 | -.071 | -.140 | .106 |
| | | Sig. (2-tailed) | .130 | .001 | .222 | .718 | .049 | .745 | .552 | .237 | .373 |
| | | N | 73 | 72 | 73 | 73 | 73 | 73 | 73 | 73 | 73 |
| | ΔGlobal ECV (ms) | Correlation Coefficient | -.023 | .486** | .158 | .320** | -.078 | -.413** | -.341** | .062 | -.052 |
| | | Sig. (2-tailed) | .854 | .000 | .199 | .008 | .525 | .000 | .004 | .614 | .672 |
| | | N | 68 | 67 | 68 | 68 | 68 | 68 | 68 | 68 | 68 |
| | ΔCD8 count (cells/μl) | Correlation Coefficient | -.018 | .060 | .065 | .045 | .135 | .077 | .272* | -.055 | .151 |
| | | Sig. (2-tailed) | .883 | .619 | .586 | .703 | .256 | .516 | .020 | .646 | .203 |
| | | N | 73 | 72 | 73 | 73 | 73 | 73 | 73 | 73 | 73 |
| | ΔCreatinine (μmol/l) | Correlation Coefficient | .148 | -.090 | .034 | -.090 | -.145 | .272* | -.138 | -.035 | -.731** |
| | | Sig. (2-tailed) | .213 | .456 | .779 | .450 | .224 | .021 | .247 | .770 | .000 |
| | | N | 72 | 71 | 72 | 72 | 72 | 72 | 72 | 72 | 72 |
| | Δhigh sensitivity CRP (mg/l) | Correlation Coefficient | .003 | .243* | .182 | .348** | -.045 | -.091 | -.138 | .338** | -.053 |
| | | Sig. (2-tailed) | .982 | .040 | .124 | .003 | .703 | .446 | .245 | .003 | .658 |
| | | N | 73 | 72 | 73 | 73 | 73 | 73 | 73 | 73 | 73 |

**Correlations**

| | | | ΔHIV viral load (log copies/ml) | ΔCD4 count (cells/μl) | ΔLV EF (%) | ΔLV EDV for height (ml/m) | ΔSTIR SIR | ΔEGE SIR | ΔGlobal native T1 (ms) | ΔGlobal T2 (ms) | ΔGlobal ECV (ms) |
|---|---|---|---|---|---|---|---|---|---|---|---|
| Spearman's rho | Δhs-cTnT (ng/l) | Correlation Coefficient | .246* | -.114 | -.062 | -.091 | .076 | -.017 | .075 | .179 | -.023 |
| | | Sig. (2-tailed) | .036 | .339 | .604 | .445 | .525 | .888 | .532 | .130 | .854 |
| | | N | 73 | 73 | 73 | 73 | 72 | 72 | 72 | 73 | 68 |
| | ΔNT-proBNP (ng/l) | Correlation Coefficient | .278* | -.130 | -.129 | .224 | -.036 | .097 | .478** | .382** | .486** |
| | | Sig. (2-tailed) | .018 | .275 | .279 | .058 | .765 | .423 | .000 | .001 | .000 |
| | | N | 72 | 72 | 72 | 72 | 71 | 71 | 71 | 72 | 67 |
| | ΔsST2 (ng/ml) | Correlation Coefficient | .189 | -.086 | -.194 | -.375** | -.012 | -.086 | .152 | -.145 | .158 |
| | | Sig. (2-tailed) | .109 | .470 | .100 | .001 | .920 | .471 | .203 | .222 | .199 |
| | | N | 73 | 73 | 73 | 73 | 72 | 72 | 72 | 73 | 68 |
| | ΔGalectin-3 (ng/ml) | Correlation Coefficient | .321** | -.128 | -.193 | -.322** | .031 | -.025 | .520** | .043 | .320** |
| | | Sig. (2-tailed) | .006 | .280 | .101 | .005 | .796 | .832 | .000 | .718 | .008 |
| | | N | 73 | 73 | 73 | 73 | 72 | 72 | 72 | 73 | 68 |
| | ΔEthanol (units/week) | Correlation Coefficient | .080 | .008 | .016 | .028 | .140 | .167 | .024 | -.232* | -.078 |
| | | Sig. (2-tailed) | .504 | .947 | .893 | .813 | .241 | .161 | .840 | .049 | .525 |
| | | N | 73 | 73 | 73 | 73 | 72 | 72 | 72 | 73 | 68 |
| | ΔWeight (kg) | Correlation Coefficient | -.180 | .197 | .063 | .322** | .059 | -.002 | -.252* | -.039 | -.413** |
| | | Sig. (2-tailed) | .129 | .095 | .596 | .005 | .620 | .988 | .032 | .745 | .000 |

| | | | | | | | | | | |
|---|---|---|---|---|---|---|---|---|---|---|
| | N | 73 | 73 | 73 | 73 | 72 | 72 | 72 | 73 | 68 |
| ΔMean arterial pressure (mmHg) | Correlation Coefficient | -.222 | .059 | .041 | .370** | -.055 | .083 | -.206 | -.071 | -.341** |
| | Sig. (2-tailed) | .059 | .620 | .728 | .001 | .646 | .486 | .082 | .552 | .004 |
| | N | 73 | 73 | 73 | 73 | 72 | 72 | 72 | 73 | 68 |
| ΔHeart rate (beats/min) | Correlation Coefficient | .248* | -.378** | -.130 | -.541** | -.173 | -.385** | .469** | -.140 | .062 |
| | Sig. (2-tailed) | .034 | .001 | .274 | .000 | .146 | .001 | .000 | .237 | .614 |
| | N | 73 | 73 | 73 | 73 | 72 | 72 | 72 | 73 | 68 |
| ΔeGFR (ml/min/1.73m $^2$) | Correlation Coefficient | -.011 | .019 | .143 | .261* | .029 | -.011 | -.060 | .106 | -.052 |
| | Sig. (2-tailed) | .924 | .875 | .228 | .026 | .810 | .925 | .620 | .373 | .672 |
| | N | 73 | 73 | 73 | 73 | 72 | 72 | 72 | 73 | 68 |
| ΔHIV viral load (log copies/ml) | Correlation Coefficient | 1.000 | -.367** | -.231* | -.146 | -.034 | .054 | .391** | .295* | .267* |
| | Sig. (2-tailed) | . | .001 | .050 | .217 | .779 | .654 | .001 | .011 | .028 |
| | N | 73 | 73 | 73 | 73 | 72 | 72 | 72 | 73 | 68 |
| ΔCD4 count (cells/μl) | Correlation Coefficient | -.367** | 1.000 | .107 | .162 | .232 | .131 | -.282* | -.049 | -.033 |
| | Sig. (2-tailed) | .001 | . | .370 | .172 | .050 | .271 | .017 | .678 | .792 |
| | N | 73 | 73 | 73 | 73 | 72 | 72 | 72 | 73 | 68 |
| ΔLV EF (%) | Correlation Coefficient | -.231* | .107 | 1.000 | .023 | -.133 | .001 | -.166 | -.313** | .036 |
| | Sig. (2-tailed) | .050 | .370 | . | .848 | .266 | .993 | .163 | .007 | .773 |
| | N | 73 | 73 | 73 | 73 | 72 | 72 | 72 | 73 | 68 |
| ΔLV EDV for height (ml/m) | Correlation Coefficient | -.146 | .162 | .023 | 1.000 | .047 | .181 | -.139 | .191 | -.099 |
| | Sig. (2-tailed) | .217 | .172 | .848 | . | .697 | .127 | .244 | .106 | .420 |
| | N | 73 | 73 | 73 | 73 | 72 | 72 | 72 | 73 | 68 |
| ΔSTIR SIR | Correlation Coefficient | -.034 | .232 | -.133 | .047 | 1.000 | .284* | -.020 | .153 | -.023 |
| | Sig. (2-tailed) | .779 | .050 | .266 | .697 | . | .017 | .868 | .200 | .851 |
| | N | 72 | 72 | 72 | 72 | 72 | 71 | 71 | 72 | 67 |
| ΔEGE SIR | Correlation Coefficient | .054 | .131 | .001 | .181 | .284* | 1.000 | -.196 | .026 | .001 |
| | Sig. (2-tailed) | .654 | .271 | .993 | .127 | .017 | . | .101 | .829 | .995 |
| | N | 72 | 72 | 72 | 72 | 71 | 72 | 71 | 72 | 68 |
| ΔGlobal native T1 (ms) | Correlation Coefficient | .391** | -.282* | -.166 | -.139 | -.020 | -.196 | 1.000 | .426** | .565** |
| | Sig. (2-tailed) | .001 | .017 | .163 | .244 | .868 | .101 | . | .000 | .000 |
| | N | 72 | 72 | 72 | 72 | 71 | 71 | 72 | 72 | 68 |
| ΔGlobal T2 (ms) | Correlation Coefficient | .295* | -.049 | -.313** | .191 | .153 | .026 | .426** | 1.000 | .343** |
| | Sig. (2-tailed) | .011 | .678 | .007 | .106 | .200 | .829 | .000 | . | .004 |
| | N | 73 | 73 | 73 | 73 | 72 | 72 | 72 | 73 | 68 |
| ΔGlobal ECV (ms) | Correlation Coefficient | .267* | -.033 | .036 | -.099 | -.023 | .001 | .565** | .343** | 1.000 |
| | Sig. (2-tailed) | .028 | .792 | .773 | .420 | .851 | .995 | .000 | .004 | . |
| | N | 68 | 68 | 68 | 68 | 67 | 68 | 68 | 68 | 68 |
| ΔCD8 count (cells/μl) | Correlation Coefficient | .142 | .256* | .069 | .243* | -.007 | .110 | -.152 | .020 | -.232 |
| | Sig. (2-tailed) | .232 | .029 | .561 | .038 | .953 | .359 | .204 | .869 | .057 |
| | N | 73 | 73 | 73 | 73 | 72 | 72 | 72 | 73 | 68 |
| ΔCreatinine (μmol/l) | Correlation Coefficient | -.096 | -.067 | -.135 | -.057 | .006 | -.015 | -.071 | .004 | -.105 |
| | Sig. (2-tailed) | .421 | .579 | .258 | .633 | .960 | .904 | .558 | .976 | .398 |
| | N | 72 | 72 | 72 | 72 | 71 | 71 | 71 | 72 | 67 |
| Δhigh sensitivity CRP (mg/l) | Correlation Coefficient | .091 | -.096 | .034 | -.178 | -.114 | -.178 | .525** | .122 | .334** |
| | Sig. (2-tailed) | .444 | .419 | .777 | .132 | .341 | .135 | .000 | .304 | .005 |
| | N | 73 | 73 | 73 | 73 | 72 | 72 | 72 | 73 | 68 |

**Correlations**

| | | | ΔCD8 count (cells/μl) | ΔCreatinine (μmol/l) | Δhigh sensitivity CRP (mg/l) |
|---|---|---|---|---|---|
| Spearman's rho | Δhs-cTnT (ng/l) | Correlation Coefficient | -.018 | .148 | .003 |
| | | Sig. (2-tailed) | .883 | .213 | .982 |
| | | N | 73 | 72 | 73 |
| | ΔNT-proBNP (ng/l) | Correlation Coefficient | .060 | -.090 | .243* |
| | | Sig. (2-tailed) | .619 | .456 | .040 |
| | | N | 72 | 71 | 72 |
| | ΔsST2 (ng/ml) | Correlation Coefficient | .065 | .034 | .182 |
| | | Sig. (2-tailed) | .586 | .779 | .124 |
| | | N | 73 | 72 | 73 |
| | ΔGalectin-3 (ng/ml) | Correlation Coefficient | .045 | -.090 | .348** |
| | | Sig. (2-tailed) | .703 | .450 | .003 |
| | | N | 73 | 72 | 73 |
| | ΔEthanol (units/week) | Correlation Coefficient | .135 | -.145 | -.045 |
| | | Sig. (2-tailed) | .256 | .224 | .703 |
| | | N | 73 | 72 | 73 |
| | ΔWeight (kg) | Correlation Coefficient | .077 | .272* | -.091 |
| | | Sig. (2-tailed) | .516 | .021 | .446 |
| | | N | 73 | 72 | 73 |
| | ΔMean arterial pressure (mmHg) | Correlation Coefficient | .272* | -.138 | -.138 |
| | | Sig. (2-tailed) | .020 | .247 | .245 |
| | | N | 73 | 72 | 73 |
| | ΔHeart rate (beats/min) | Correlation Coefficient | -.055 | -.035 | .338** |
| | | Sig. (2-tailed) | .646 | .770 | .003 |
| | | N | 73 | 72 | 73 |
| | ΔeGFR (ml/min/1.73m$^2$) | Correlation Coefficient | .151 | -.731** | -.053 |
| | | Sig. (2-tailed) | .203 | .000 | .658 |
| | | N | 73 | 72 | 73 |
| | ΔHIV viral load (log copies/ml) | Correlation Coefficient | .142 | -.096 | .091 |
| | | Sig. (2-tailed) | .232 | .421 | .444 |
| | | N | 73 | 72 | 73 |
| | ΔCD4 count (cells/μl) | Correlation Coefficient | .256* | -.067 | -.096 |
| | | Sig. (2-tailed) | .029 | .579 | .419 |
| | | N | 73 | 72 | 73 |
| | ΔLV EF (%) | Correlation Coefficient | .069 | -.135 | .034 |
| | | Sig. (2-tailed) | .561 | .258 | .777 |
| | | N | 73 | 72 | 73 |
| | ΔLV EDV for height (ml/m) | Correlation Coefficient | .243* | -.057 | -.178 |
| | | Sig. (2-tailed) | .038 | .633 | .132 |
| | | N | 73 | 72 | 73 |
| | ΔSTIR SIR | Correlation Coefficient | -.007 | .006 | -.114 |
| | | Sig. (2-tailed) | .953 | .960 | .341 |
| | | N | 72 | 71 | 72 |

| | | | | | |
|---|---|---|---|---|---|
| | ΔEGE SIR | Correlation Coefficient | .110 | -.015 | -.178 |
| | | Sig. (2-tailed) | .359 | .904 | .135 |
| | | N | 72 | 71 | 72 |
| | ΔGlobal native T1 (ms) | Correlation Coefficient | -.152 | -.071 | .525** |
| | | Sig. (2-tailed) | .204 | .558 | .000 |
| | | N | 72 | 71 | 72 |
| | ΔGlobal T2 (ms) | Correlation Coefficient | .020 | .004 | .122 |
| | | Sig. (2-tailed) | .869 | .976 | .304 |
| | | N | 73 | 72 | 73 |
| | ΔGlobal ECV (ms) | Correlation Coefficient | -.232 | -.105 | .334** |
| | | Sig. (2-tailed) | .057 | .398 | .005 |
| | | N | 68 | 67 | 68 |
| | ΔCD8 count (cells/μl) | Correlation Coefficient | 1.000 | -.225 | -.246* |
| | | Sig. (2-tailed) | . | .057 | .036 |
| | | N | 73 | 72 | 73 |
| | ΔCreatinine (μmol/l) | Correlation Coefficient | -.225 | 1.000 | .055 |
| | | Sig. (2-tailed) | .057 | . | .646 |
| | | N | 72 | 72 | 72 |
| | Δhigh sensitivity CRP (mg/l) | Correlation Coefficient | -.246* | .055 | 1.000 |
| | | Sig. (2-tailed) | .036 | .646 | . |
| | | N | 73 | 72 | 73 |

*. Correlation is significant at the 0.05 level (2-tailed).

**. Correlation is significant at the 0.01 level (2-tailed).

hs-cTnT = high sensitivity cardiac troponin T; NT-proBNP = N-terminal pro B-type natriuretic peptide; sST2 = soluble ST2; CRP = C-reactive protein; eGFR = estimated glomerular filtration rate; LA = left atrium; RA = right atrium; LV = left ventricle; EDV = end diastolic volume; STIR = short tau inversion recovery; SIR = skeletal muscle to myocardial signal intensity ratio; EGE = early gadolinium enhancement; RV = right ventricle; EF = ejection fraction; ECV = extracellular volume mapping

# Supplement B: Bivariate correlations within the HIV-infected group (baseline and follow up)

**Correlations**

| | | | hs-cTnT (ng/l) | NT-proBNP (ng/l) | sST2 (ng/ml) | Galectin-3 (ng/ml) | Mean Arterial Pressure (mmHg) | Weight (kg) | 6-Minute walk test - distance (m) | High sensitivity CRP (mg/l) | CD4 count (cells/µl) | HIV viral load (log copies/ml) |
|---|---|---|---|---|---|---|---|---|---|---|---|---|
| Spearman's rho | hs-cTnT (ng/l) | Correlation Coefficient | 1.000 | .018 | .293** | .036 | -.015 | .157 | .106 | .105 | -.126 | .062 |
| | | Sig. (2-tailed) | . | .830 | .000 | .667 | .857 | .059 | .204 | .206 | .129 | .455 |
| | | N | 146 | 145 | 146 | 146 | 146 | 146 | 146 | 146 | 146 | 146 |
| | NT-proBNP (ng/l) | Correlation Coefficient | .018 | 1.000 | .076 | .357** | -.147 | -.262** | -.217** | .275** | -.165* | .232** |
| | | Sig. (2-tailed) | .830 | . | .362 | .000 | .078 | .001 | .009 | .001 | .047 | .005 |
| | | N | 145 | 145 | 145 | 145 | 145 | 145 | 145 | 145 | 145 | 145 |
| | sST2 (ng/ml) | Correlation Coefficient | .293** | .076 | 1.000 | .451** | -.106 | -.151 | .049 | .193* | -.034 | .189* |
| | | Sig. (2-tailed) | .000 | .362 | . | .000 | .203 | .068 | .558 | .020 | .681 | .022 |
| | | N | 146 | 145 | 146 | 146 | 146 | 146 | 146 | 146 | 146 | 146 |
| | Galectin-3 (ng/ml) | Correlation Coefficient | .036 | .357** | .451** | 1.000 | -.183* | -.282** | -.133 | .277** | -.276** | .188* |
| | | Sig. (2-tailed) | .667 | .000 | .000 | . | .027 | .001 | .110 | .001 | .001 | .023 |
| | | N | 146 | 145 | 146 | 146 | 146 | 146 | 146 | 146 | 146 | 146 |
| | Mean Arterial Pressure (mmHg) | Correlation Coefficient | -.015 | -.147 | -.106 | -.183* | 1.000 | .364** | .170* | -.166* | .212* | -.058 |
| | | Sig. (2-tailed) | .857 | .078 | .203 | .027 | . | .000 | .040 | .045 | .010 | .485 |
| | | N | 146 | 145 | 146 | 146 | 146 | 146 | 146 | 146 | 146 | 146 |
| | Weight (kg) | Correlation Coefficient | .157 | -.262** | -.151 | -.282** | .364** | 1.000 | .178* | -.150 | .178* | -.145 |
| | | Sig. (2-tailed) | .059 | .001 | .068 | .001 | .000 | . | .031 | .071 | .031 | .081 |
| | | N | 146 | 145 | 146 | 146 | 146 | 146 | 146 | 146 | 146 | 146 |
| | 6-Minute walk test - distance (m) | Correlation Coefficient | .106 | -.217** | .049 | -.133 | .170* | .178* | 1.000 | -.105 | .049 | -.019 |
| | | Sig. (2-tailed) | .204 | .009 | .558 | .110 | .040 | .031 | . | .209 | .555 | .821 |
| | | N | 146 | 145 | 146 | 146 | 146 | 146 | 146 | 146 | 146 | 146 |
| | High sensitivity CRP (mg/l) | Correlation Coefficient | .105 | .275** | .193* | .277** | -.166* | -.150 | -.105 | 1.000 | -.212* | .281** |
| | | Sig. (2-tailed) | .206 | .001 | .020 | .001 | .045 | .071 | .209 | . | .010 | .001 |
| | | N | 146 | 145 | 146 | 146 | 146 | 146 | 146 | 146 | 146 | 146 |
| | CD4 count (cells/µl) | Correlation Coefficient | -.126 | -.165* | -.034 | -.276** | .212* | .178* | .049 | -.212* | 1.000 | -.425** |
| | | Sig. (2-tailed) | .129 | .047 | .681 | .001 | .010 | .031 | .555 | .010 | . | .000 |
| | | N | 146 | 145 | 146 | 146 | 146 | 146 | 146 | 146 | 146 | 146 |
| | HIV viral load (log copies/ml) | Correlation Coefficient | .062 | .232** | .189* | .188* | -.058 | -.145 | -.019 | .281** | -.425** | 1.000 |
| | | Sig. (2-tailed) | .455 | .005 | .022 | .023 | .485 | .081 | .821 | .001 | .000 | . |
| | | N | 146 | 145 | 146 | 146 | 146 | 146 | 146 | 146 | 146 | 146 |

| | | | | | | | | | | | |
|---|---|---|---|---|---|---|---|---|---|---|---|
| eGFR (ml/min/1.73m$^2$) | Correlation Coefficient | -.055 | .007 | -.100 | -.263** | .253** | .461** | -.056 | -.069 | .108 | .146 |
| | Sig. (2-tailed) | .510 | .929 | .230 | .001 | .002 | .000 | .502 | .408 | .194 | .079 |
| | N | 146 | 145 | 146 | 146 | 146 | 146 | 146 | 146 | 146 | 146 |
| Creatinine (μmol/l) | Correlation Coefficient | .357** | -.259** | .169* | .078 | .000 | .275** | .383** | -.050 | .015 | -.246** |
| | Sig. (2-tailed) | .000 | .002 | .042 | .347 | .999 | .001 | .000 | .547 | .861 | .003 |
| | N | 146 | 145 | 146 | 146 | 146 | 146 | 146 | 146 | 146 | 146 |
| LA area (cm2) | Correlation Coefficient | .123 | .044 | .151 | -.034 | .205* | .383** | .339** | -.021 | .210* | -.209* |
| | Sig. (2-tailed) | .140 | .602 | .069 | .685 | .013 | .000 | .000 | .801 | .011 | .011 |
| | N | 146 | 145 | 146 | 146 | 146 | 146 | 146 | 146 | 146 | 146 |
| RA area (cm2) | Correlation Coefficient | .316** | -.080 | .144 | -.194* | .230** | .497** | .442** | -.012 | .226** | -.159 |
| | Sig. (2-tailed) | .000 | .338 | .083 | .019 | .005 | .000 | .000 | .886 | .006 | .055 |
| | N | 146 | 145 | 146 | 146 | 146 | 146 | 146 | 146 | 146 | 146 |
| LV mass for height (g/m) | Correlation Coefficient | .314** | -.179* | .303** | -.081 | .311** | .382** | .432** | .044 | .168* | -.026 |
| | Sig. (2-tailed) | .000 | .031 | .000 | .329 | .000 | .000 | .000 | .595 | .043 | .751 |
| | N | 146 | 145 | 146 | 146 | 146 | 146 | 146 | 146 | 146 | 146 |
| LV EDV for height (ml/m) | Correlation Coefficient | .310** | -.003 | .253** | -.116 | .146 | .313** | .435** | -.036 | .165* | -.163* |
| | Sig. (2-tailed) | .000 | .974 | .002 | .162 | .078 | .000 | .000 | .666 | .046 | .049 |
| | N | 146 | 145 | 146 | 146 | 146 | 146 | 146 | 146 | 146 | 146 |
| RV EDV for height (ml/m) | Correlation Coefficient | .292** | -.057 | .272** | -.136 | .172* | .301** | .407** | -.008 | .238** | -.125 |
| | Sig. (2-tailed) | .000 | .494 | .001 | .102 | .038 | .000 | .000 | .928 | .004 | .134 |
| | N | 146 | 145 | 146 | 146 | 146 | 146 | 146 | 146 | 146 | 146 |
| LV EF (%) | Correlation Coefficient | -.122 | -.036 | -.113 | -.122 | .073 | .025 | -.124 | -.013 | .065 | -.016 |
| | Sig. (2-tailed) | .143 | .669 | .175 | .142 | .384 | .767 | .136 | .872 | .436 | .846 |
| | N | 146 | 145 | 146 | 146 | 146 | 146 | 146 | 146 | 146 | 146 |
| RV EF (%) | Correlation Coefficient | -.104 | .076 | -.126 | -.013 | -.085 | .002 | -.098 | -.085 | -.011 | -.099 |
| | Sig. (2-tailed) | .212 | .365 | .129 | .880 | .310 | .984 | .238 | .309 | .894 | .236 |
| | N | 146 | 145 | 146 | 146 | 146 | 146 | 146 | 146 | 146 | 146 |
| Global T1 Time Average (ms) | Correlation Coefficient | -.288** | .402** | -.115 | .245** | -.243** | -.348** | -.401** | .266** | -.219** | .311** |
| | Sig. (2-tailed) | .000 | .000 | .167 | .003 | .003 | .000 | .000 | .001 | .008 | .000 |
| | N | 145 | 144 | 145 | 145 | 145 | 145 | 145 | 145 | 145 | 145 |
| Global T2 average (ms) | Correlation Coefficient | -.053 | .465** | -.016 | .290** | -.244** | -.285** | -.252** | .154 | -.209* | .126 |
| | Sig. (2-tailed) | .528 | .000 | .847 | .000 | .003 | .000 | .002 | .064 | .011 | .129 |
| | N | 146 | 145 | 146 | 146 | 146 | 146 | 146 | 146 | 146 | 146 |
| Global ECV average (ms) | Correlation Coefficient | -.113 | .440** | .064 | .274** | -.216* | -.385** | -.421** | .274** | -.221** | .232** |

| | | | | | | | | | | | |
|---|---|---|---|---|---|---|---|---|---|---|---|
| | | Sig. (2-tailed) | .181 | .000 | .452 | .001 | .010 | .000 | .000 | .001 | .008 | .006 |
| | | N | 141 | 140 | 141 | 141 | 141 | 141 | 141 | 141 | 141 | 141 |

**Correlations**

| | | | eGFR (ml/min/1.73 $m^2$) | Creatinine (μmol/l) | LA area (cm2) | RA area (cm2) | LV mass for height (g/m) | LV EDV for height (ml/m) | RV EDV for height (ml/m) | LV EF (%) | RV EF (%) | Global native T1 (ms) |
|---|---|---|---|---|---|---|---|---|---|---|---|---|
| Spearman's rho | hs-cTnT (ng/l) | Correlation Coefficient | -.055 | .357** | .123 | .316** | .314** | .310** | .292** | -.122 | -.104 | -.288** |
| | | Sig. (2-tailed) | .510 | .000 | .140 | .000 | .000 | .000 | .000 | .143 | .212 | .000 |
| | | N | 146 | 146 | 146 | 146 | 146 | 146 | 146 | 146 | 146 | 145 |
| | NT-proBNP(ng/l) | Correlation Coefficient | .007 | -.259** | .044 | -.080 | -.179* | -.003 | -.057 | -.036 | .076 | .402** |
| | | Sig. (2-tailed) | .929 | .002 | .602 | .338 | .031 | .974 | .494 | .669 | .365 | .000 |
| | | N | 145 | 145 | 145 | 145 | 145 | 145 | 145 | 145 | 145 | 144 |
| | sST2 (ng/ml) | Correlation Coefficient | -.100 | .169* | .151 | .144 | .303** | .253** | .272** | -.113 | -.126 | -.115 |
| | | Sig. (2-tailed) | .230 | .042 | .069 | .083 | .000 | .002 | .001 | .175 | .129 | .167 |
| | | N | 146 | 146 | 146 | 146 | 146 | 146 | 146 | 146 | 146 | 145 |
| | Galectin-3 (ng/ml) | Correlation Coefficient | -.263** | .078 | -.034 | -.194* | -.081 | -.116 | -.136 | -.122 | -.013 | .245** |
| | | Sig. (2-tailed) | .001 | .347 | .685 | .019 | .329 | .162 | .102 | .142 | .880 | .003 |
| | | N | 146 | 146 | 146 | 146 | 146 | 146 | 146 | 146 | 146 | 145 |
| | Mean Arterial Pressure (mmHg) | Correlation Coefficient | .253** | .000 | .205* | .230** | .311** | .146 | .172* | .073 | -.085 | -.243** |
| | | Sig. (2-tailed) | .002 | .999 | .013 | .005 | .000 | .078 | .038 | .384 | .310 | .003 |
| | | N | 146 | 146 | 146 | 146 | 146 | 146 | 146 | 146 | 146 | 145 |
| | Weight (kg) | Correlation Coefficient | .461** | .275** | .383** | .497** | .382** | .313** | .301** | .025 | .002 | -.348** |
| | | Sig. (2-tailed) | .000 | .001 | .000 | .000 | .000 | .000 | .000 | .767 | .984 | .000 |
| | | N | 146 | 146 | 146 | 146 | 146 | 146 | 146 | 146 | 146 | 145 |
| | 6-Minute walk test - distance (m) | Correlation Coefficient | -.056 | .383** | .339** | .442** | .432** | .435** | .407** | -.124 | -.098 | -.401** |
| | | Sig. (2-tailed) | .502 | .000 | .000 | .000 | .000 | .000 | .000 | .136 | .238 | .000 |
| | | N | 146 | 146 | 146 | 146 | 146 | 146 | 146 | 146 | 146 | 145 |
| | High sensitivity CRP (mg/l) | Correlation Coefficient | -.069 | -.050 | -.021 | -.012 | .044 | -.036 | -.008 | -.013 | -.085 | .266** |
| | | Sig. (2-tailed) | .408 | .547 | .801 | .886 | .595 | .666 | .928 | .872 | .309 | .001 |
| | | N | 146 | 146 | 146 | 146 | 146 | 146 | 146 | 146 | 146 | 145 |
| | CD4 count (cells/μl) | Correlation Coefficient | .108 | .015 | .210* | .226** | .168* | .165* | .238** | .065 | -.011 | -.219** |
| | | Sig. (2-tailed) | .194 | .861 | .011 | .006 | .043 | .046 | .004 | .436 | .894 | .008 |
| | | N | 146 | 146 | 146 | 146 | 146 | 146 | 146 | 146 | 146 | 145 |
| | HIV viral load (log copies/ml) | Correlation Coefficient | .146 | -.246** | -.209* | -.159 | -.026 | -.163* | -.125 | -.016 | -.099 | .311** |
| | | Sig. (2-tailed) | .079 | .003 | .011 | .055 | .751 | .049 | .134 | .846 | .236 | .000 |
| | | N | 146 | 146 | 146 | 146 | 146 | 146 | 146 | 146 | 146 | 145 |

| | | | | | | | | | | | | |
|---|---|---|---|---|---|---|---|---|---|---|---|---|
| | Absolute eGFR (ml/min/1.73m$^2$) | Correlation Coefficient | 1.000 | -.517** | .128 | .124 | .133 | .106 | .068 | .086 | .174* | -.014 |
| | | Sig. (2-tailed) | . | .000 | .125 | .137 | .109 | .202 | .413 | .303 | .035 | .866 |
| | | N | 146 | 146 | 146 | 146 | 146 | 146 | 146 | 146 | 146 | 145 |
| | Creatinine (μmol/l) | Correlation Coefficient | -.517** | 1.000 | .267** | .382** | .392** | .361** | .352** | -.208* | -.219** | -.459** |
| | | Sig. (2-tailed) | .000 | . | .001 | .000 | .000 | .000 | .000 | .012 | .008 | .000 |
| | | N | 146 | 146 | 146 | 146 | 146 | 146 | 146 | 146 | 146 | 145 |
| | LA area (cm2) | Correlation Coefficient | .128 | .267** | 1.000 | .638** | .585** | .676** | .669** | -.041 | -.106 | -.227** |
| | | Sig. (2-tailed) | .125 | .001 | . | .000 | .000 | .000 | .000 | .624 | .203 | .006 |
| | | N | 146 | 146 | 146 | 146 | 146 | 146 | 146 | 146 | 146 | 145 |
| | RA area (cm2) | Correlation Coefficient | .124 | .382** | .638** | 1.000 | .696** | .734** | .779** | -.002 | -.185* | -.441** |
| | | Sig. (2-tailed) | .137 | .000 | .000 | . | .000 | .000 | .000 | .981 | .025 | .000 |
| | | N | 146 | 146 | 146 | 146 | 146 | 146 | 146 | 146 | 146 | 145 |
| | LV mass for height (g/m) | Correlation Coefficient | .133 | .392** | .585** | .696** | 1.000 | .739** | .780** | -.092 | -.282** | -.357** |
| | | Sig. (2-tailed) | .109 | .000 | .000 | .000 | . | .000 | .000 | .272 | .001 | .000 |
| | | N | 146 | 146 | 146 | 146 | 146 | 146 | 146 | 146 | 146 | 145 |
| | LV EDV for height (ml/m) | Correlation Coefficient | .106 | .361** | .676** | .734** | .739** | 1.000 | .867** | -.260** | -.167* | -.354** |
| | | Sig. (2-tailed) | .202 | .000 | .000 | .000 | .000 | . | .000 | .002 | .044 | .000 |
| | | N | 146 | 146 | 146 | 146 | 146 | 146 | 146 | 146 | 146 | 145 |
| | RV EDV for height (ml/m) | Correlation Coefficient | .068 | .352** | .669** | .779** | .780** | .867** | 1.000 | -.011 | -.333** | -.386** |
| | | Sig. (2-tailed) | .413 | .000 | .000 | .000 | .000 | .000 | . | .897 | .000 | .000 |
| | | N | 146 | 146 | 146 | 146 | 146 | 146 | 146 | 146 | 146 | 145 |
| | LV EF (%) | Correlation Coefficient | .086 | -.208* | -.041 | -.002 | -.092 | -.260** | -.011 | 1.000 | .450** | -.011 |
| | | Sig. (2-tailed) | .303 | .012 | .624 | .981 | .272 | .002 | .897 | . | .000 | .899 |
| | | N | 146 | 146 | 146 | 146 | 146 | 146 | 146 | 146 | 146 | 145 |
| | RV EF (%) | Correlation Coefficient | .174* | -.219** | -.106 | -.185* | -.282** | -.167* | -.333** | .450** | 1.000 | .093 |
| | | Sig. (2-tailed) | .035 | .008 | .203 | .025 | .001 | .044 | .000 | .000 | . | .266 |
| | | N | 146 | 146 | 146 | 146 | 146 | 146 | 146 | 146 | 146 | 145 |
| | Global native T1 (ms) | Correlation Coefficient | -.014 | -.459** | -.227** | -.441** | -.357** | -.354** | -.386** | -.011 | .093 | 1.000 |
| | | Sig. (2-tailed) | .866 | .000 | .006 | .000 | .000 | .000 | .000 | .899 | .266 | . |
| | | N | 145 | 145 | 145 | 145 | 145 | 145 | 145 | 145 | 145 | 145 |
| | Global T2 average (ms) | Correlation Coefficient | -.160 | -.181* | .096 | -.166* | -.165* | -.023 | -.109 | -.151 | .042 | .522** |
| | | Sig. (2-tailed) | .054 | .029 | .249 | .046 | .046 | .782 | .189 | .070 | .611 | .000 |
| | | N | 146 | 146 | 146 | 146 | 146 | 146 | 146 | 146 | 146 | 145 |
| | Global ECV average (ms) | Correlation Coefficient | -.069 | -.407** | -.154 | -.383** | -.255** | -.250** | -.277** | .023 | .101 | .732** |

| | | | | | | | | | | |
|---|---|---|---|---|---|---|---|---|---|---|
| Sig. (2-tailed) | .418 | .000 | .069 | .000 | .002 | .003 | .001 | .783 | .232 | .000 |
| N | 141 | 141 | 141 | 141 | 141 | 141 | 141 | 141 | 141 | 141 |

**Correlations**

| | | | Global T2 (ms) | Global ECV (ms) |
|---|---|---|---|---|
| Spearman's rho | hs-cTnT (ng/l) | Correlation Coefficient | -.053 | -.113 |
| | | Sig. (2-tailed) | .528 | .181 |
| | | N | 146 | 141 |
| | NT-proBNP(ng/l) | Correlation Coefficient | .465** | .440** |
| | | Sig. (2-tailed) | .000 | .000 |
| | | N | 145 | 140 |
| | sST2 (ng/ml) | Correlation Coefficient | -.016 | .064 |
| | | Sig. (2-tailed) | .847 | .452 |
| | | N | 146 | 141 |
| | Galectin-3 (ng/ml) | Correlation Coefficient | .290** | .274** |
| | | Sig. (2-tailed) | .000 | .001 |
| | | N | 146 | 141 |
| | Mean Arterial Pressure (mmHg) | Correlation Coefficient | -.244** | -.216* |
| | | Sig. (2-tailed) | .003 | .010 |
| | | N | 146 | 141 |
| | Weight (kg) | Correlation Coefficient | -.285** | -.385** |
| | | Sig. (2-tailed) | .000 | .000 |
| | | N | 146 | 141 |
| | 6-Minute walk test - distance (m) | Correlation Coefficient | -.252** | -.421** |
| | | Sig. (2-tailed) | .002 | .000 |
| | | N | 146 | 141 |
| | High sensitivity CRP (mg/l) | Correlation Coefficient | .154 | .274** |
| | | Sig. (2-tailed) | .064 | .001 |
| | | N | 146 | 141 |
| | CD4 count (cells/μl) | Correlation Coefficient | -.209* | -.221** |
| | | Sig. (2-tailed) | .011 | .008 |
| | | N | 146 | 141 |
| | HIV viral load (log copies/ml) | Correlation Coefficient | .126 | .232** |
| | | Sig. (2-tailed) | .129 | .006 |
| | | N | 146 | 141 |
| | Absolute eGFR (ml/min/1.73m$^2$) | Correlation Coefficient | -.160 | -.069 |
| | | Sig. (2-tailed) | .054 | .418 |
| | | N | 146 | 141 |
| | Creatinine (μmol/l) | Correlation Coefficient | -.181* | -.407** |
| | | Sig. (2-tailed) | .029 | .000 |
| | | N | 146 | 141 |
| | LA area (cm2) | Correlation Coefficient | .096 | -.154 |
| | | Sig. (2-tailed) | .249 | .069 |
| | | N | 146 | 141 |
| | RA area (cm2) | Correlation Coefficient | -.166* | -.383** |
| | | Sig. (2-tailed) | .046 | .000 |

| | | | | |
|---|---|---|---|---|
| | | N | 146 | 141 |
| | LV mass for height (g/m) | Correlation Coefficient | -.165* | -.255** |
| | | Sig. (2-tailed) | .046 | .002 |
| | | N | 146 | 141 |
| | LV EDV for height (ml/m) | Correlation Coefficient | -.023 | -.250** |
| | | Sig. (2-tailed) | .782 | .003 |
| | | N | 146 | 141 |
| | RV EDV for height (ml/m) | Correlation Coefficient | -.109 | -.277** |
| | | Sig. (2-tailed) | .189 | .001 |
| | | N | 146 | 141 |
| | LV EF (%) | Correlation Coefficient | -.151 | .023 |
| | | Sig. (2-tailed) | .070 | .783 |
| | | N | 146 | 141 |
| | RV EF (%) | Correlation Coefficient | .042 | .101 |
| | | Sig. (2-tailed) | .611 | .232 |
| | | N | 146 | 141 |
| | Global T1 Time Average (ms) | Correlation Coefficient | .522** | .732** |
| | | Sig. (2-tailed) | .000 | .000 |
| | | N | 145 | 141 |
| | Global T2 average (ms) | Correlation Coefficient | 1.000 | .579** |
| | | Sig. (2-tailed) | . | .000 |
| | | N | 146 | 141 |
| | Global ECV average (ms) | Correlation Coefficient | .579** | 1.000 |
| | | Sig. (2-tailed) | .000 | . |
| | | N | 141 | 141 |

**. Correlation is significant at the 0.01 level (2-tailed).

*. Correlation is significant at the 0.05 level (2-tailed).

hs-cTnT = high sensitivity cardiac troponin T; NT-proBNP = N-terminal pro B-type natriuretic peptide; sST2 = soluble ST2; CRP = C-reactive protein; eGFR = estimated glomerular filtration rate; LA = left atrium; RA = right atrium; LV = left ventricle; EDV = end diastolic volume; RV = right ventricle; EF = ejection fraction; ECV = extracellular volume mapping
